# Case report: A novel mutation in the EYA1 gene in a child with branchiootic syndrome with secretory otitis media and bilateral vestibular hypofunction

**DOI:** 10.3389/fgene.2023.1292085

**Published:** 2024-01-08

**Authors:** Jun He, Ahmad Mahmoudi, Yu Gu, Jinfeng Fu, Qiulin Yuan, Wei Liu

**Affiliations:** ^1^ Department of Otolaryngology-Head and Neck Surgery, The Second Xiangya Hospital, Central South University, Changsha, Hunan, China; ^2^ Department of Otolaryngology-Head and Neck Surgery, Stanford University School of Medicine, Stanford, CA, United States

**Keywords:** branchiootic syndrome, *EYA1* gene mutation, whole exome sequencing, secretory otitis media, vestibular hypofunction, case report

## Abstract

Branchiootic syndrome (BOS) is a rare, autosomal dominant syndrome characterized by malformations of the ear associated with hearing loss, second branchial arch anomalies, and the absence of renal anomalies. Herein, we report the case of an 8-year-old male patient with BOS. The proband also experiences mixed conductive and sensorineural hearing loss in the right ear, and severe-to-profound sensorineural hearing loss in the left ear. Preauricular pits, branchial fistulae, and cochlear hypoplasia were present bilaterally. Type III cup-shaped ear, and external auditory canal stenosis were detected in the right ear. Lateral semicircular canal-vestibule dysplasia was detected in the left ear. Moreover, the patient had unilateral secretory otitis media (SOM) in the right ear and bilateral vestibular hypofunction (VH), which has not been reported in previous studies. The patient’s hearing on the right side was restored to nearly normal after myringotomy. Whole exome sequencing identified a novel frameshift mutation in *EYA1* (NM_000503.6): c.1697_1698delinT [p.(Lys566IlefsTer73)] in the proband, which was defined a “pathogenic” mutation according to American College of Medical Genetics and Genomics guidelines. This is the first report of a child presenting with BOS, SOM and VH, which expands the known clinical manifestations of this syndrome. We also observed a novel *EYA1* gene mutation in this patient with BOS, which enriches the mutation map and provides a reference for genetic diagnosis of this syndrome.

## 1 Introduction

Branchiootorenal syndrome (BOR1, MIM# 113650) is an autosomal dominant disorder characterized by branchiogenic malformations, various degrees of hearing loss, and renal involvement (Zheng et al., 2021). Branchiootic syndrome (BOS1, MIM# 602588) is distinguished by normal renal anatomy and function. The incidence of this disorder is approximately 1 in 40,000 live births, and accounts for about 2% of children with severe deafness (Li et al., 2018).

Molecular studies have been performed in patients with BOR/BOS, which revealed the most known causative genes were *EYA1* (BOR1, MIM# 113650, BOS1, MIM# 602588), *SIX1* (BOS3, MIM# 608389), and *SIX5* (BOR2, MIM# 610896). Of these genes, *EYA1* variants—including frameshifts, and partial duplications—account for the majority of diagnosed patients (Dantas et al., 2015; Wang et al., 2018). *EYA1* is a transcription factor important in developing several tissues, including the branchial arches, kidney, eyes, and ear. However, no significant relationship has been shown between the nature of the mutations and the clinical features associated with BOR/BOS. Whole-exome sequencing (WES) is an efficient strategy to identify the coding sequences of a genome, in which 85% of pathogenic mutations are located, and has shown significant advantages in diagnosing genetic disorders (Rabbani et al., 2014; Yang et al., 2015).

Herein, we report a Chinese child with BOS with a novel pathogenic mutation in the *EYA1* gene and secretory otitis media (SOM) and vestibular hypofunction (VH), which has not been previously reported. WES and Sanger sequencing was used to analyze the proband’s DNA sample. We conducted this study to expand both the mutational spectrum and clinical characteristics of BOS.

## 2 Materials and methods

### 2.1 Case report

**FIGURE 1:**
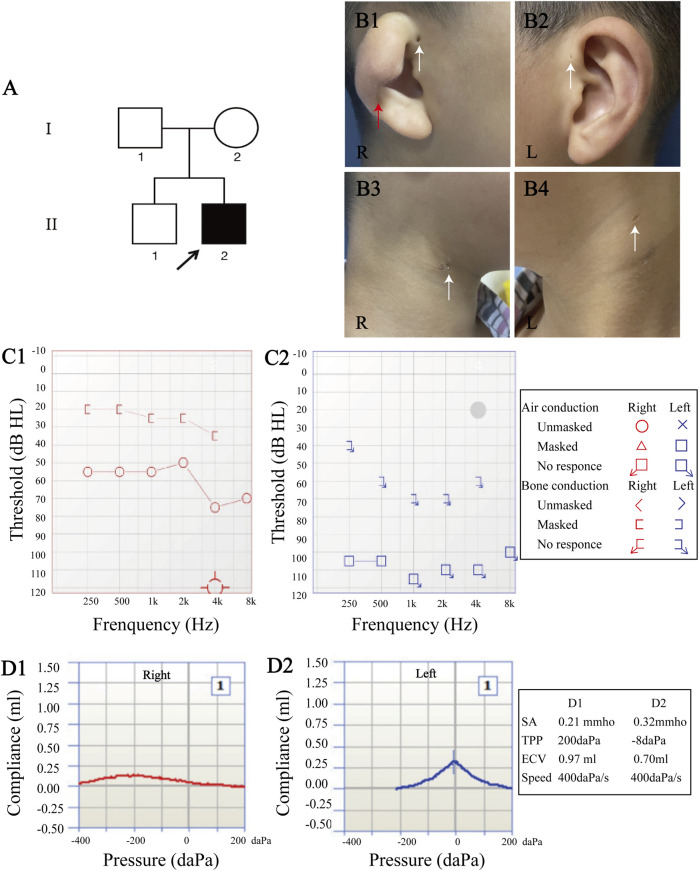
Clinical manifestations and audiological evaluation. **(A)** Pedigree of family. **(B)** The proband had type III cup-shaped right ear (red arrow **(B1)**), external auditory canal stenosis, bilateral preauricular pits (white arrows **(B1–B2)**), and bilateral branchial fistulae (white arrows **(B3–B4)**). **(C)** Pure-tone audiometry revealed mixed hearing loss in the right ear (average air conduction threshold, 59 dB; average bone conduction threshold, 26 dB **(C1)**), and severe-to-profound sensorineural hearing loss in the left ear **(C2)**. **(D)** Acoustic immittance test showed type “C” tympanogram in the right ear that indicates secretory otitis media **(D1)**, and type “A” tympanogram in the left ear **(D2)**.

An 8-year-old boy presented to The Second Xiangya Hospital of Central South University, China with bilateral hearing loss, preauricular pits, and branchial fistulae, along with an external ear anomaly in the right ear (Figure 1 B1-B4). The patient also had SOM in the right ear, which caused ear fullness and further mixed hearing loss. After extensive audiological and radiological evaluations, the patient, who has no past medical history, was diagnosed with BOS. He and his family then enrolled in our study to undergo further evaluations (Figures 1A–Ⅱ:2).

**FIGURE 2:**
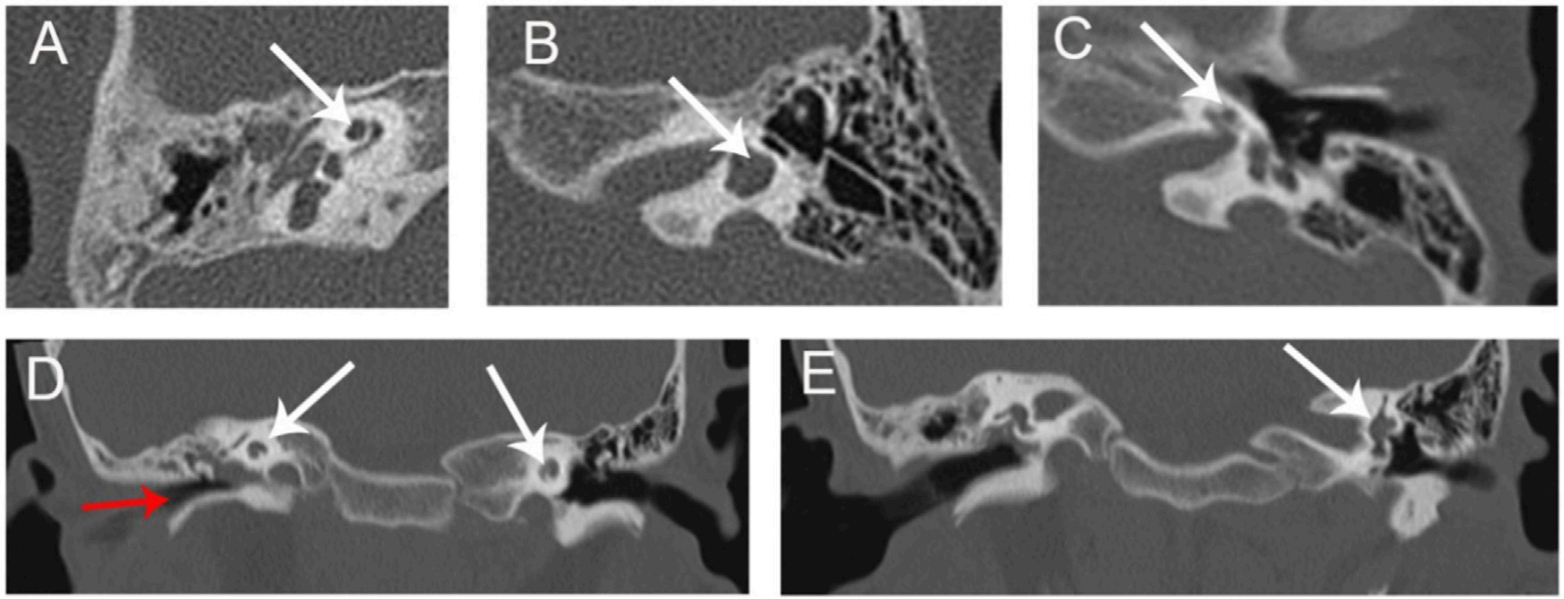
HRCT of proband **(A)** Cochlear hypoplasia in the right ear (arrow). **(B)** Lateral semicircular canal-vestibule dysplasia in the left ear (arrow). **(C)** Cochlear hypoplasia with less than two turns in the left ear. **(D)** Soft tissue shadow in the right middle ear (red arrow). Cochlear hypoplasia on both sides (white arrows). **(E)** Lateral semicircular canal-vestibule dysplasia in the left ear (arrow). **(A–C)** Coronal view; **(D,E)** Axial view. HRCT, high-resolution computed tomography.

Micro-otoscopy and pure-tone audiometry (PTA) were used to evaluate hearing levels (Figure 1 C1-C2). High-resolution computed tomography (HRCT) of the temporal bone was performed to determine the middle and inner ear morphologies (Figure 2). Renal examinations, including renal ultrasound, renal function tests, and urinalysis were performed to screen for renal abnormalities. He subsequently underwent myringotomy with grommet insertion at otolaryngology ward of the Second Xiangya Hospital. Three months after surgery, an objective audiometry comprised of acoustic immittance, distortion product otoacoustic emission, auditory brainstem response (ABR), and auditory steady-state response were conducted. Vestibular function and vestibular evoked myogenic potential (VEMP) were also assessed.

### 2.2 Genomic DNA extraction and quality assessment

Peripheral blood samples were collected from the proband and his family members. Genomic DNA was extracted using a DNA extraction kit (Agilent Technologies), and the DNA quality of the samples was controlled using a NanoDrop 2000 Spectrophotometer (ThermoFisher Scientific). Purified DNA was quantified using Qubit (ThermoFisher Scientific).

### 2.3 Library construction, capture, and WES

The SureSelect Human All Exon V6 capture system (Agilent Technologies) was used for library construction and capture. The sequencing data were obtained in FASTQ format by high-throughput sequencing using the 2 × 150 bp double-terminal sequencing mode.

### 2.4 Data screening and bioinformatics analysis

After quality control, the FASTQ data were aligned to hg38 (GCF_000001405.26) from the National Center for Biotechnology Information (https://www.ncbi.nlm.nih.gov) by using Burrows-Wheeler Aligner 6. GATK software (Broad Institute) was used to determine single nucleotide variants (SNVs) and small fragment insertion-deletion mutations (InDels). Data filtering was completed using the dbSNP, ExAC03, HapMap, and 1,000 Genomes databases, and sites with a mutation frequency of less than 1% were retained. The mutation site with the highest priority was defined as “First1” and was considered as the candidate gene. Finally, we used Phenolyzer software (http://phenolyzer.wglab.org/) to predict gene pathogenicity of the candidate gene in BOS.

### 2.5 Sanger sequencing

Candidate mutation was validated using Sanger sequencing to verify the DNA sequence variants detected by WES. Primers were designed for the target region using Primer3 online software (http://bioinfo.ut.ee/primer3-0.4.0/). The forward and reverse primers were F: 5-CTG​CAC​ATA​TTC​ATC​ACG​TTT​CAC​A-3; and R: 5-CAC​TAG​GAA​AAG​AAA​GCT​GTT​TTG​AGA​G-3, respectively. After purification with shrimp alkaline phosphatase and exonuclease I (Agilent Technologies), the PCR products were sequenced on an ABI 3730XL DNA Analyzer (ThermoFisher Scientific). The sequence reads were analyzed using the PolyPhred software (https://droog.gs.washington.edu/polyphred/).

## 3 Results

### 3.1 Clinical manifestations and family members characteristics

Medical history and physical examination of the family revealed that only the child with BOS presented with preauricular pits, branchial fistulae, type III cup-shaped ear, external auditory canal stenosis, inner ear anomaly, and hearing loss (Figures 1, 2). This was the first time that hearing loss was diagnosed in the patient, and no previous ear related intervention had been performed. The proband and his family members had no history of using ototoxic drug or noise exposure. The preoperative assessment results of the proband were as follows; pure-tone audiometry showed mixed hearing loss in the right ear, where the average air and bone conduction thresholds were 59 and 26 dB, respectively, and severe-to-profound sensorineural hearing loss in the left ear (Figure 1 C1-C2). Acoustic immittance test showed type “A” tympanogram in the left ear and type “C” tympanogram in the right ear that indicated SOM (Figure 1 D1-D2). Acoustic reflex thresholds could not be obtained in both ears through 500–4,000 Hz. Videonystagmography revealed normal ocular, positional, Dix-Hallpike and roll tests, ruling out a diagnosis of benign paroxysmal positional vertigo. The caloric test revealed bilateral hyporeflexia with reflexes of 2.6 deg/s and 5.0 deg/s after cool water stimulation, and 5.5 deg/s and 4.5 deg/s after warm water stimulation, on the right and left sides, respectively. VEMP were absent after stimulation on both sides. In the right inner ear, HRCT of the temporal bone indicated soft tissue shadow in the right middle ear and cochlear hypoplasia. In the left inner ear, HRCT revealed cochlear hypoplasia and lateral semicircular canal-vestibule (Figure 2). Abdominal color doppler ultrasound, and routine urine and blood biochemical profiles showed no abnormalities. The proband underwent myringotomy with grommet insertion in the right ear, during which viscous fluid accumulation was visible in the middle ear. Consequently, the postoperative ABR was 30 dBnHL in the right ear, indicating a significant improvement compared to the preoperative value.

### 3.2 Genetic and molecular analysis

#### 3.2.1 Quality control of WES

After quality assessment of the raw data and comparative analysis with reference sequence (hg38), we obtained the following quality assessment data. On average, we generated >12 G of total data per sample. The average ratio of the base Q30 was >91%, and the average mean coverage sequencing depth for the intended target was 106X. When measured at 20X depth, 97% of the target was covered. These results suggest that the data captured by WES is adequate for reliably detecting DNA variants for further analysis.

#### 3.2.2 Gene screening and validation of mutation sites

**FIGURE 3:**
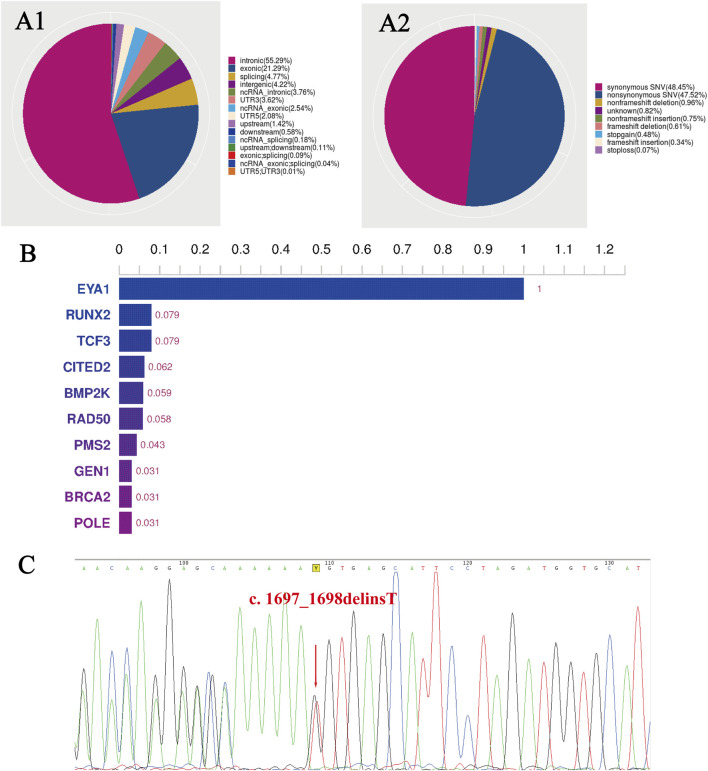
The WES results of the family with BOS. **(A1)** Distribution ratio of SNVs/InDels sites in different gene regions, and **(A2)** of different functional types. **(B)** The histogram of the top 10 genes. The *EYA1* gene has the highest score. **(C)** A sequencing chromatography of the reverse complementary strand of the frameshift variant in the proband. The red arrow indicates a change in base position, and the 73rd codon, followed by lysine (Lys566), has become a stop codon. WES, whole-exome sequencing; BOS, branchiootic syndrome; SNVs, single nucleotide variants; InDels, insertion-deletion mutations.

Mutation sites were classified according to their position relative to the gene regions (Figure 3A1) and functional positions (Figure 3A2). Based on the software analysis results, we got the top 10 genes. The *EYA1* gene has the highest score (Figure 3B).

Bioinformatic analysis of the proband’s WES revealed a heterozygous mutation in. *EYA1* (NM_000503.6): c.1697_1698delinT [p.(Lys566IlefsTer73)] at exon 17, where the 566th amino acid changed from lysine to isoleucine. This variant was not found in dbSNP, ExAC03, HapMap, HGMD, or 1,000 Genomes databases and did not exist in the average population. To verify the WES results, we performed Sanger sequencing on all family members and observed that only the proband had the *EYA1* gene c.1697_1698delinsT mutation (Figure 3C), whereas other healthy family members had no mutation at this site.

## 4 Discussion

BOS is a rare autosomal dominant disorder with high genotype heterogeneity (Chen et al., 2019). It is expressed differently in various individuals, and currently, there is no evidence of genotype-phenotype correlations in patient with BOS (Unzaki et al., 2018). In 2004, Chang et al. (2004) proposed a widely accepted diagnostic criteria for BOR/BOS using the major and minor criteria. The major criteria include branchial arch anomalies, hearing loss, preauricular pits, and renal anomalies; minor criteria include external, middle, and inner ear anomalies, preauricular tags, facial asymmetry, and palatal abnormalities (Table 1). Patients without a family history are diagnosed with BOR if they meet three or more of the above major criteria or two major and at least two minor criteria (Chang et al., 2004). A previous study reported the frequency of major criteria in BOR as follows: hearing loss 95.4%, preauricular pits 87%, branchial arch anomalies 86.5%, and renal anomalies 58.3% (Li et al., 2020). From a clinical standpoint, hearing loss is the most common and constant feature of the *EYA1* gene mutations and is detected in more than 90% of affected individuals (Chang et al., 2004). Treatment of BOS is mainly symptomatic, including the surgical removal of branchial arch fistulae and cochlear implants (Men et al., 2020).

**TABLE 1:** Clinical characteristics in BOR/BOS.

	Clinical manifestations	BOR^a^	BOS^a^	Our patient
Major Criteria	Hearing Loss	✓	✓	✓
	Periauricular Pits	✓	✓	✓
	Branchial Arch Anomalies	✓	✓	✓
	Renal Anomalies	✓		
Minor Criteria	External Ear Anomalies	✓	✓	✓
	Middle Ear Anomalies	✓	✓	
	Inner Ear Anomalies	✓	✓	✓
	Periauricular Tags	✓	✓	
	Facial Asymmetry, Palate Abnormalities	✓	✓	
New Findings	Secretory Otitis Media			✓
	Vestibular Hypofunction			✓

BOR, branchiootorenal syndrome; BOS, branchiootic syndrome.

^a^ Affected individuals must meet at least three major criteria, two major criteria and at least two minor criteria, or one major criterion and an affected first-degree relative.

The proband in this study had known BOS clinical presentations such as bilateral preauricular pits, bilateral cervical branchial arch fistulae, hearing loss, right external ear, and bilateral inner ear anomalies. In addition to the above manifestations, the patient had SOM and bilateral VH, phenotypes not previously reported in other studies. While SOM in an 8-year-old child is not rare, SOM in the proband occurred on the same side as the external ear anomaly. This indicates that BOS may affect the Eustachian tube and ear ventilation, which can lead to inflammation. The proband also has VH, which has not previously been associated with BOS. The most common etiology of bilateral VH is gentamicin ototoxicity, followed by other rare entities, such as autoimmune inner ear disease, meningitis, Meniere’s disease and vestibular neuritis; these features were not present in the proband (Hain et al., 2018). HRCT indicated lateral semicircular canal-vestibule dysplasia in the proband’s left ear (Figures 2B, E), which may have caused VH. This finding warrants more attention to the vestibular functions in BOS patients in the future.

Nowadays, WES can sensitively detect sequence variants. In this study, we identified a novel *EYA1* frameshift mutation c.1697_1698delinsT [p.(Lys566IlefsTer73)] using read-depth analysis of WES data in this family with BOS and verified by Sanger sequencing in the proband but not in unaffected family members. The *EYA1* gene, located on chromosome 8q13.3, is the human homolog of the *Drosophila EYA1* gene, which is essential for eye development in this species (Orten et al., 2008). The EYA1 protein is also important for the ear, branchial arches, and kidney development. Animal model studies have shown the *EYA1* homozygous-deficient mice lack ears and kidneys, and the *EYA1* heterozygous-deficient mice present phenotypes resembling BOR (Xu et al., 1999; Xu et al., 2002). The known pathogenic genes of BOR/BOS include *EYA1*, *SIX1*, and *SIX5*. The *EYA1* gene mutations account for approximately 40% of BOR cases, while *SIX5* and *SIX1* genes account for 5% and less than 1%, respectively (Shah et al., 2020). To date, more than 240 pathological mutations in the *EYA1* gene have been reported. In China, *EYA1* c.466C>T [p.(Gln156Ter)], and *EYA1* c.1735delG [p.(Asp579 fs)] were the first variants of *EYA1* identified in 2012 (Wang et al., 2012). With the development of next-generation sequencing technology, an increasing number of novel *EYA1* mutations have been reported in Chinese patients with BOS/BOR (Table 2). Han et al. (2021) recruited 7 members of a Chinese family, 4 of whom affected with BOS. *EYA1* c.1627C>T [p.(Gln543Ter)] was identified as a novel mutation and pathogenic cause of BOS. Similarly, Xing et al. (2020) also collected blood samples from 6 members of a Chinese family, 4 of whom affected by BOS, and found a novel frameshift variant, c.1075_1077delinsAT [p.(Gly359 fs)] in the *EYA1* gene. These results suggest that the BOR/BOS criteria are potential indications for molecular studies to diagnose *EYA1*-associated syndromes (Chen et al., 2019; Li et al., 2020).

**TABLE 2:** Reported *EYA1* gene mutations in Chinese patients with BOR/BOS.

Phenotype	Location	Mutation	Protein change	Mutation type	References
BOR	Exon 10	c.889C>T	p.(Arg297Ter)	Nonsense	Feng et al. (2021)
BOR	Exon 11	c.967A>T	p.(Arg323Ter)	Nonsense	Wang et al. (2018)
BOR	Exon 7	c.466C>T	p.(Gln156Ter)	Nonsense	Wang et al. (2012)
BOS	Exon 17	c.1627C>T	p.(Gln543Ter)	Nonsense	Han et al. (2021)
BOS	Intron 10	c.967-2A>G	——	Splicing	Chen et al. (2019)
BOR	Intron 11	c.1050 + 1G>T	——	Splicing	Feng et al. (2021)
BOR	Intron 12	c.1140 + 1G>A	——	Splicing	Feng et al. (2021)
BOS	Exon 12	c.1075_1077delinsAT	p.(Gly359 fs)	Frameshift	Xing et al. (2020)
BOR	Exon 15	c.1381delA	p.(Arg461 fs)	Frameshift	Li et al. (2018)
BOR	Exon 15	c.1425delC	p.(Asp579 fs)	Frameshift	Feng et al. (2021)
BOS	Exon 16	c.1493_1494insAT	p.(Ile498 fs)	Frameshift	Chen et al. (2019)
BOR	Exon 17	c.1735delG	p.(Asp579 fs)	Frameshift	Wang et al. (2012)
BOR	Exons 1–18	Entire deletion	Loss protein	CNVs	Zheng et al. (2021)
BOR	Exons 1–18	Entire deletion	Loss protein	CNVs	Han et al. (2021)

*EYA1*, eyes absent homolog 1; BOR, branchiootorenal syndrome; BOS, branchiootic syndrome; CNVs, copy number variations.

*EYA1* is also affected by gene dose effects, and clinical phenotypic heterogeneity may be related to insufficient *EYA1* gene dosage. However, gene activity can be recognized only when the number of encoded proteins exceeds a certain threshold. In addition, gene regulation of specific molecules that encode different amounts of gene products explains the wide phenotypic variation in BOS patients between or within families (Wang et al., 2018).

Based on the American College of Medical Genetics and Genomics (ACMG) guidelines, the following three criteria were applied to interpret this *EYA1* c.1697_1698delinsT as a pathogenic variant with an important role in *EYA1*-assoicated conditions. First, it is a frameshift mutation in the *EYA1* and causes gene loss-of-function; this is a known disease mechanism (very strong pathogenic criterion; PVS1). The absence of this variation in the proband’s parents suggests that this is a *de novo* mutation (strong pathogenic criterion; PS2). Finally, since this variant was not found in the Genome Aggregation Database (gnomAD), the rarity of the mutation was recognized (moderate pathogenic criterion; PM2).

This *de novo* mutation in our proband contributes to an autosomal dominant disorder. According to the law of autosomal dominant inheritance, it is estimated that the offsprings of the proband have a 50% increased risk of inheriting *EYA1* pathogenic variants. Therefore, detecting the pathogenic variants for this disease is of great importance, and patients suspected of BOS should be monitored accordingly for associated otologic and second branchial arch anomalies. With the emergence of new sequencing technologies in recent years, many novel pathogenic mutations have been reported, but many unknown pathogenic variants still need to be discovered. The novel *EYA1* frameshift mutation discovered in this study can be equip BOS genetic testing in the future for early diagnose of diseases and to assist with the personalized prenatal screening.

## 5 Conclusion

We identified a novel heterozygous *de novo* pathogenic frameshift mutation in the *EYA1* gene in a proband with BOS. Previously unreported clinical manifestations of BOS, including bilateral vestibular hypofunction and unilateral secretory otitis media, were observed. Our results expand both the mutational and phenotypic spectra of BOS, suggesting that the mutation of a key amino acid is an etiological factor. Considering the high phenotypic heterogeneity of BOS in patients whose diagnosis is difficult based on clinical manifestations alone, genetic testing for candidate pathogenic gene mutations can successfully complement clinical diagnosis.

## Data Availability

The datasets presented in this study can be found in online repositories. The names of the repository/repositories and accession number(s) can be found below: GEO database and the accession number is PRJNA979267.

## References

[B1] Chang E. H. Menezes M. Meyer N. C. Cucci R. A. Vervoort V. S. Schwartz C. E. (2004). Branchio-oto-renal syndrome: the mutation spectrum in EYA1 and its phenotypic consequences. Hum. Mutat. 23 (6), 582–589. 10.1002/humu.20048 15146463

[B2] Chen P. Liu H. Lin Y. Xu J. Zhu W. Wu H. (2019). EYA1 mutations leads to Branchio-Oto syndrome in two Chinese Han deaf families. Int. J. Pediatr. otorhinolaryngology 123, 141–145. 10.1016/j.ijporl.2019.05.006 31102969

[B3] Dantas V. G. Freitas E. L. Della-Rosa V. A. Lezirovitz K. de Moraes A. M. S. M. Ramos S. B. (2015). Novel partial duplication of EYA1 causes branchiootic syndrome in a large Brazilian family. Int. J. audiology 54 (9), 593–598. 10.3109/14992027.2015.1030511 25926005

[B4] Feng H. Xu H. Chen B. Sun S. Zhai R. Zeng B. (2021). Genetic and phenotypic variability in Chinese patients with branchio-oto-renal or branchio-oto syndrome. Front. Genet. 12, 765433. 10.3389/fgene.2021.765433 34868248 PMC8634836

[B5] Hain T. C. Cherchi M. Yacovino D. A. (2018). Bilateral vestibular weakness. Front. neurology 9, 344. 10.3389/fneur.2018.00344 PMC5990606 29904366

[B6] Han R. Xia Y. Liu Z. Wu S. Ye E. Duan L. (2021). A mutation of EYA1 gene in a Chinese Han family with Branchio-Oto syndrome. Medicine 100 (25), e24691. 10.1097/MD.0000000000024691 34160378 PMC8238333

[B7] Li G. Shen Q. Sun L. Liu H. An Y. Xu H. (2018). A *de novo* and novel mutation in the EYA1 gene in a Chinese child with branchio-oto-renal syndrome. Intractable rare Dis. Res. 7 (1), 42–45. 10.5582/irdr.2017.01075 29552445 PMC5849624

[B8] Li H. X. Zhou P. Tong M. Zheng Y. (2020). Branchial cleft fistula to branchio-oto-renal syndrome: a case report and literature review. J. Int. Med. Res. 48 (7), 300060520926363. 10.1177/0300060520926363 32689865 PMC7375735

[B9] Men M. Li W. Chen H. Wu J. Feng Y. Guo H. (2020). Identification of a novel CNV at 8q13 in a family with branchio-oto-renal syndrome and epilepsy. Laryngoscope 130 (2), 526–532. 10.1002/lary.27941 30908667

[B10] Orten D. J. Fischer S. M. Sorensen J. L. Radhakrishna U. Cremers C. W. R. J. Marres H. A. M. (2008). Branchio-oto-renal syndrome (BOR): novel mutations in the EYA1 gene, and a review of the mutational genetics of BOR. Hum. Mutat. 29 (4), 537–544. 10.1002/humu.20691 18220287

[B11] Rabbani B. Tekin M. Mahdieh N. (2014). The promise of whole-exome sequencing in medical genetics. J. Hum. Genet. 59 (1), 5–15. 10.1038/jhg.2013.114 24196381

[B12] Shah A. M. Krohn P. Baxi A. B. Tavares A. L. P. Sullivan C. H. Chillakuru Y. R. (2020). Six1 proteins with human branchio-oto-renal mutations differentially affect cranial gene expression and otic development. Dis. models Mech. 13 (3), dmm043489. 10.1242/dmm.043489 PMC7063838 31980437

[B13] Unzaki A. Morisada N. Nozu K. Ye M. J. Ito S. Matsunaga T. (2018). Clinically diverse phenotypes and genotypes of patients with branchio-oto-renal syndrome. J. Hum. Genet. 63 (5), 647–656. 10.1038/s10038-018-0429-8 29500469

[B14] Wang S. H. Wu C. C. Lu Y. C. Lin Y. H. Su Y. N. Hwu W. L. (2012). Mutation screening of the EYA1, SIX1, and SIX5 genes in an East Asian cohort with branchio-oto-renal syndrome. Laryngoscope 122 (5), 1130–1136. 10.1002/lary.23217 22447252

[B15] Wang Y. G. Sun S. P. Qiu Y. L. Xing Q. H. Lu W. (2018). A novel mutation in EYA1 in a Chinese family with Branchio-oto-renal syndrome. BMC Med. Genet. 19 (1), 139. 10.1186/s12881-018-0653-2 30086703 PMC6081847

[B16] Xing Z. K. Wang S. Y. Xia X. Ding W. J. Duan L. Cui X. (2020). Targeted next-generation sequencing identifies a novel frameshift EYA1 variant causing branchio-otic syndrome in a Chinese family. Int. J. Pediatr. otorhinolaryngology 138, 110202. 10.1016/j.ijporl.2020.110202 32717629

[B17] Xu P. X. Adams J. Peters H. Brown M. C. Heaney S. Maas R. (1999). Eya1-deficient mice lack ears and kidneys and show abnormal apoptosis of organ primordia. Nat. Genet. 23 (1), 113–117. 10.1038/12722 10471511

[B18] Xu P. X. Zheng W. Laclef C. Maire P. Maas R. L. Peters H. (2002). Eya1 is required for the morphogenesis of mammalian thymus, parathyroid and thyroid. Dev. Camb. Engl. 129 (13), 3033–3044. 10.1242/dev.129.13.3033 PMC3873877 12070080

[B19] Yang H. Robinson P. N. Wang K. (2015). Phenolyzer: phenotype-based prioritization of candidate genes for human diseases. Nat. Methods 12, 841–843. 10.1038/nmeth.3484 26192085 PMC4718403

[B20] Zheng H. Xu J. Wang Y. Lin Y. Hu Q. Li X. (2021). Identification and characterization of a cryptic genomic deletion-insertion in EYA1 associated with branchio-otic syndrome. Neural plast. 2021, 5524381. 10.1155/2021/5524381 33880118 PMC8046558

