# Supplementary material for: Case report: A novel mutation in the EYA1 gene in a child with branchiootic syndrome with secretory otitis media and bilateral vestibular hypofunction
Source: Front Genet. 2024 Jan 8;14:1292085. doi: 10.3389/fgene.2023.1292085 (PMC10801012; doi:10.3389/fgene.2023.1292085)
Supplement: Supplementary file 1 [file Table1.docx]

**The EYA1 gene reference sequence:**

>8_71210657_71211657

GAGAGAGACAGGTAGGACCTCATGATATGAAGAAGACATCGAGTCTTCTGGGGCAGCAGATAGTTTTATCTTCCAAGTCAGCTTTGACCAACTGATAGAGGCTTTCTGAAGGATGTTTGAAAGGTTCTTAGGCTGCGTCCAGGCTCAGGTAACAAGGTTTGAACCACCTGGCATGTCTGCCATGGGTACAGAAAAGGTCCTGCTGGCACATGTGTTATCTCTGCTCTGGATGACAGCTACACCACTAACAACACTTCAGTTCTGCTCCCAAATTCTACCTTCTATGAACACCACTTGTGGCCCTTGAGTTTGTGCACACTACTATTCAGAAAGATCACTCCATATTTATTGAATTCTACTTTACCCTGTTCAGTGCTTAGAGTACTGCACATATTCATCACGTTTCACATAAATGGAACCTGTTTAAATGATCCAGTTATGAGAATACTGAGGACTGAAAAAACAAATGAGACAAGATGCACCATCTAGGAATGCTCACCTTTTTTGCTCCTTGTTCTTCTTCTACACCATCTCCTATAACAACATACACCACTTTTCTTCCAAACCTTTGAATTATTCTCTCAAAACAGCTTTCTTTTCCTAGTGAACAAAAATAAATGATAGAAAATGTGAAGTTTGGGTAACCTAATGTGACAGTGCTTGAACTTTTTATATAAAATGCTTTCTTCATGCTCTGATTCGAATGCCCCACTAGTACCTCTAAGAGGAATGCTTTTATGCCACTTGGGTTGTATGTGCCACAGCACAATAAATTACAAAGTAAATTAATTAGCAGTGTGGTGTCCTGTCCTTTGGAAATTGATAGTTAAATACTCTCTTCCCAAGGACTCATTTATTAAAAAATGGAATCTAGTCAATCACAGCATTGCAATTGGGGTGGTGGGGGAAAGTACTGTTTTGCAGTGGGACTTCTTTTTCCCCCTATATATAAGCTGAACTCAAACATAGGCAATTCTGGCAACATATTTTAACTTGATTTT
